# Supplementary material for: The Ghost of Predator Past: Interaction of Past Predator Exposure and Resource Availability on Toxin Retention and Cell Growth in a Dinoflagellate
Source: Toxins (Basel). 2025 Jun 7;17(6):290. doi: 10.3390/toxins17060290 (PMC12197344; doi:10.3390/toxins17060290)
Supplement: Supplementary file 1 [file toxins-17-00290-s001.zip › toxins-3607482-supplementary.pdf]

Supplementary Materials

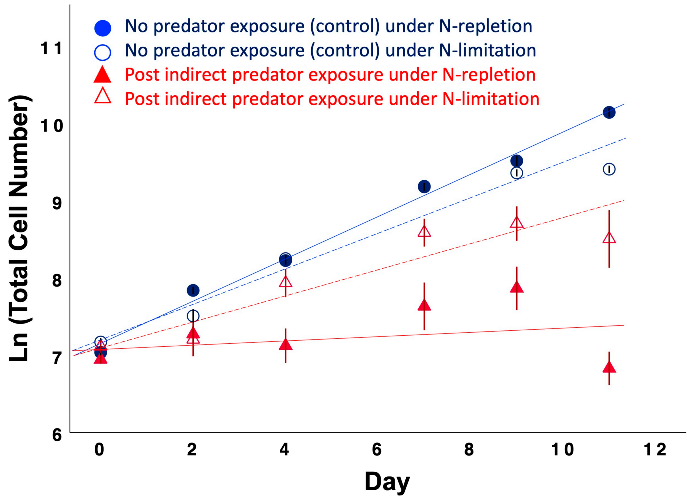

Figure S1. Growth curves of *Alexandrium catenella*, past indirectly-induced by predators compared to control cells which were not induced. Growth rates of treatment and control cells were also compared in N-replete (f/2) and N-limited (filtered seawater) media. Regression of the natural log of total cell number versus time represents the average growth rate (d^{-1}) of cells of the past grazer exposed treatments (n=10) and controls (n=10). Bars represent ±1 standard error of the mean number of cells of 10 replicates for each treatment or control at each time point.

Table S1. Summary of two-way ANOVA for cell growth rate (d^{-1}) from Assay 1. Factors in the ANOVA are past predator exposure (G), nitrogen (N), and their interaction. SS(III), type III sum of squares; df, degrees of freedom; MS, mean sum of squares for ANOVA; *F*, statistic for ANOVA test; *p*, significance of the ANOVA test; and η^2, partial eta squared.

Cell growth rate						
Source	SS(III)	df	MS	*F*	*p*	η^2
Grazing	0.32	1	0.319	54	<.001	0.60
Nitrogen	0.01	1	0.003	0.52	0.474	0.01
G × N	0.11	1	0.107	18	<.001	0.34
Error	0.21	36	0.006			
Total	1.49	40				

Table S2. Summary of two-way ANOVA for dependent variables: Cell toxin content (fmol cell^{-1}) and cell growth rate (d^{-1}) from the exponential phase (EP). Factors in the ANOVA are past predator exposure (G), Media (M), and their interaction. SS(III), type III sum of squares; df, degrees of freedom; MS, mean sum of squares for ANOVA; *F*, statistic for ANOVA test; *p*, significance of the ANOVA test; and η^2, partial eta squared.

Cell toxin content						
Source	SS(III)	df	MS	*F*	*p*	η^2
Grazing	20202	2	10101	440	<.001	0.98
Media	2399	5	479	21	<.001	0.85
G × M	1250	10	125	5	<.001	0.75
Error	412	18	22			
Total	86349	36				

Cell growth rate						
Source	SS(III)	df	MS	*F*	*p*	η^2
Grazing	0.10	2	0.050	48	<.001	0.57
Media	0.01	5	0.001	1.28	0.280	0.08
G × M	0.02	10	0.002	2.03	0.042	0.22
Error	0.07	72	0.001			
Total	0.37	90				

Table S3. Summary of two-way ANOVA for dependent variables: Cell toxin content (fmol cell^{-1}) and cell growth rate (d^{-1}) from the stationary phase (SP). Factors in the ANOVA are past predator exposure (G), Media (M), and their interaction. SS(III), type III sum of squares; df, degrees of freedom; MS, mean sum of squares for ANOVA; *F*, statistic for ANOVA test; *p*, significance of the ANOVA test; and η^2, partial eta squared.

Cell toxin content						
Source	SS(III)	df	MS	*F*	*p*	η^2
Grazing	10810	2	5405	372	<.001	0.98
Media	2856	5	571	39	<.001	0.92
G × M	1128	10	112	7.77	<.001	0.81
Error	261	18	14.5			
Total	115229	36				
Cell growth rate						
Source	SS(III)	df	MS	*F*	*p*	η^2
Grazing	0.09	2	0.045	27.5	<.001	0.43
Media	0.01	5	0.001	0.57	0.726	0.04
G × M	0.01	10	0.001	0.68	0.740	0.09
Error	0.12	72	0.002			
Total	0.42	90				

Table S4. Summary of two-way ANOVA for dependent variables: Cell toxin content (fmol cell^{-1}) and cell growth rate (d^{-1}) from the declining phase (DP). Factors in the ANOVA are past predator exposure (G), Media (M), and their interaction. SS(III), type III sum of squares; df, degrees of freedom; MS, mean sum of squares for ANOVA; *F*, statistic for ANOVA test; *p*, significance of the ANOVA test; and η^2, partial eta squared.

Cell toxin content						
Source	SS(III)	df	MS	*F*	*p*	η^2
Grazing	738	2	369	27.3	<.001	0.75
Media	128	5	26	1.91	0.143	0.35
G × M	1071	10	107	7.94	<.001	0.82
Error	243	18	14			
Total	34222	36				
**Cell growth rate**						
Source	SS(III)	df	MS	*F*	*p*	η^2
Grazing	0.03	2	0.014	9.26	<.001	0.21
Media	0.01	5	0.001	0.74	0.594	0.05
G × M	0.01	10	0.001	0.75	0.673	0.10
Error	0.10	69	0.002			
Total	0.18	87				

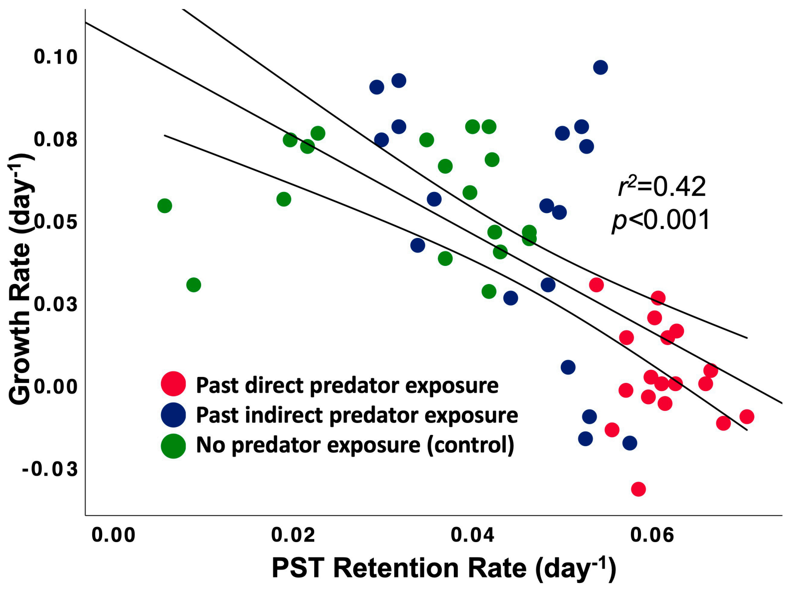

Figure S2. A tradeoff between PST retention rate (exponential) vs cell growth rate (A). Lines represent the best fit regression of PST retention rate versus growth rate with 95% confidence intervals.

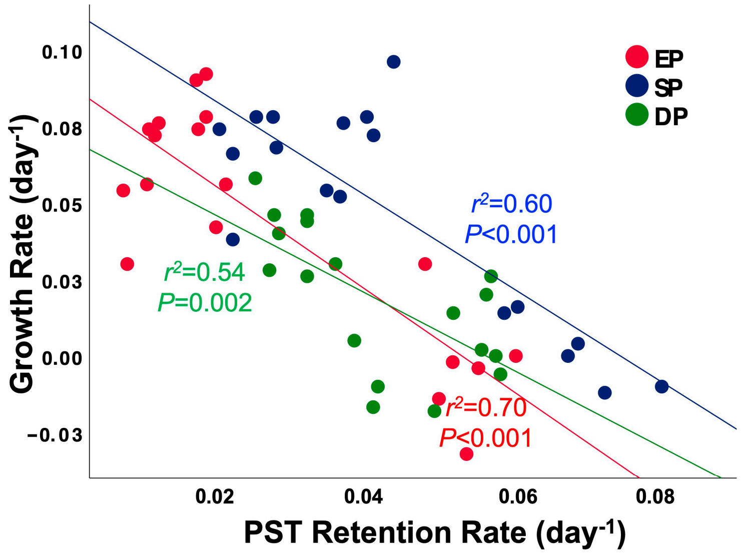

Figure S3. Tradeoffs between PST retention rate (linear) vs cell growth rate for the exponential phase (EP), stationary phase (SP), and declining phase (DP). Lines represent the best fit regression of PST retention rate versus growth rate.
